# The Noncompliant Patient

**DOI:** 10.14797/mdcvj.1166

**Published:** 2022-12-06

**Authors:** James B. Young, Justin C. Cordova

**Affiliations:** 1 Emeritus Executive Director of Academic Affairs, Cleveland Clinic, Professor of Medicine, Cleveland Clinic Lerner College of Medicine of Case Western Reserve University, Cleveland, Ohio, US; 2 Department of Anesthesiology, Walter Reed National Military Medical Center, Bethesda, Maryland, US

## Abstract

This issue’s Poet’s Pen is an experiential work submitted by Captain Justin C. Cordova, MD, from the Department of Anesthesiology at Walter Reed National Military Medical Center in Bethesda, Maryland. The submission was a response to an invitation for our readers to submit poems for consideration of publication. Captain Cordova’s work, “Against Medical Advice,” is an engaging and provocative poem that focuses on a disturbing healthcare provider challenge—attempting to deliver “best care” to patients who, for countless reasons, choose a pathway we label as noncompliance or against medical advice.

## Against Medical Advice

Desperately wanting to treat him, we found ourselves begging him to stay.

Every morning, I’d turn on his lights and ask permission

 

to cure the death silently festering inside of him.

This man was in his forties and he’d simply had enough

 

of drains and operations, of treatments and procedures.

His abdomen was infiltrated by abscesses, plethoric

 

opacities visible only through computed tomography,

treatable only by explicit acquiescence. Without it

 

this infection, the source of his unbreakable fever,

this stairway that elevated his leukocytes beyond the grasp

 

of our most aggressive antibiotics,

would undoubtedly be the cause of his demise.

 

We longed to treat him, this man who lived in a park

more than 30 miles from our hospital.

 

We warned and pleaded, implored and argued;

he wanted only to return to his version of home.

 

We could not force him to stay, could not coerce his consent

for life-giving treatment. Instead we searched for a surrogate, a

 

second best to the standard of care. We patched him up and loaded him down,

ensuring that his ill-advised transition would at least be well-equipped,

 

providing for him the complex medical supplies

required to care for his wounds and adding our prayers

 

as an honorific to his sacred autonomy.

We knew we’d see him again, just hoped he’d be alive when we did,

 

hoped he could stay the oars of Charon just long enough

for us to find him and finally treat his infection.

 

I was supremely overjoyed to see him return, alive, just four days later;

the pain from his infection was nearing a zenith of unbearability,

 

his wounds oozing far beyond the control of his portable pharmacy.

I marshalled my emotions, strictly avoiding every hint of remonstration,

 

but contrition was clearly marked upon every crease of his sun-darkened face,

his very reappearance lending weight to the expanse of our admonitions.

 

In this, our penultimate encounter, he finally surrendered to our advice, allowing

the interventional radiologists to place the drains that would save his future,

 

sheepish and apologetic in his faithful adherence to our every suggestion.

Yes, I was thankful to see this patient comply to our wishes;

 

yes, I was grateful for this belated deference to expertise and experience;

yet, above all, I found myself most thankful that he was still alive.

 

CPT Justin C. Cordova, MC USA

Walter Reed National Military Medical Center

## Commentary

William Carlos Williams, an iconic American poet and physician, wrote in his introduction to Allen Ginsberg’s *Howl & Other Poems*, “Poets are damned but they are not blind, they see with the eyes of the angels.”^1^ This issue’s Poet’s Pen is an experiential work submitted by Captain Justin C. Cordova from the Department of Anesthesiology at Walter Reed National Military Medical Center in Bethesda, Maryland. The submission was a response to an invitation for our readers to submit poems for consideration of publication.

Captain Cordova’s work, “Against Medical Advice,” is an engaging and provocative poem that focuses on a disturbing healthcare provider challenge—attempting to deliver “best care” to patients who, for countless reasons, choose a pathway we label noncompliant or against medical advice. At times it is catastrophic, yet common to encounter. It can be as simple as not stopping smoking, not dieting for weight control and diabetes management, or refusing to get a COVID-19 vaccination. More problematic is patient noncompliance when they are referred for major life-saving surgical or device-based interventions.

There are differences, however, between a noncompliant patient who intentionally refuses to follow treatment recommendations and a nonadherent patient who unintendedly does not comply with taking medications or refuses prescribed treatments—the former situation being most difficult. Over the past decades, a massive amount of work has been done analyzing this complex challenge.^1,2^ In a seminal paper on this subject, Donavan and Blake posited that “The solution to the waste of resources inherent in non-compliance lies not in attempting to increase patient adherence *per se* but in the development of more open, co-operative doctor-patient relationships.” This includes, in my opinion, taking the stance of an angel’s nonjudgmental eye and view.

“Against Medical Advice” is a story about the dance caregivers use to achieve best therapeutic outcomes in noncompliant patients going against medical advice. Many, such as the Cordova patient, are homeless and have significant social and mental health problems that influence behavior, which we try to address. The poem is skillfully crafted with a message about the doctor-patient relationship and the power of what Donavan and Blake referred to as developing a “co-operative” doctor-patient relationship.^3^ The poem is unfettered by restraints of rhyme, rhythm, or format and is simply a provocative free-verse story. The lines remind me of some of the Beat Poets—Gertrude Stein’s (pre-Beat generation) “Stanzas,” Allen Ginsberg’s “Howl,” Jack Kerouac’s “On the Road,” and Lawrence Ferlinghetti’s “A Coney Island of the Mind.” Free-verse stories carrying a punch.

Reading “Against Medical Advice” reminded me of interactions with my own noncompliant patients over the past 50 years. Over time I learned that anger, frustration, remonstration, and paternalism were not appropriate responses. Rather, nonjudgmental acceptance of a patient’s decision was the first step in building a relationship that might, in the end, allow a challenging patient to see the rationale and wisdom of a caregiver’s recommendation and plan. I have seen that happen often.

The poem also reminded me of a similar patient from 50 years ago who I followed during my clinical training and then as a Staff Physician at Houston’s Ben Taub General Hospital. He was constantly being readmitted after refusing longer stays in extended-care rehabilitation facilities. Delirium tremens, episodes of sepsis, diabetic ketoacidosis, congestive heart failure, and decubitus ulcers were devastating, but the patient lived beyond expectations. He was in and out of most available Harris County extended-care facilities but always left prematurely after getting well enough to walk. His departure was invariably against medical advice. I would get a call whenever he returned and was re-hospitalized. But then the calls stopped. I learned he died in a homeless camp.

I remember vividly the last conversation we had after he’d sobered up following one of his numerous delirium tremens admissions. All he did, for the first time, was thank me and the team that cared for him over the years, reassuring us that he was happy while he asked for our understanding. For the first time, he apologized for being unable to follow our recommendations and plans. He simply could not stop smoking, stop drinking, or stay out of the hospital. Of course, he had mental health issues that seemed untreatable. But he appeared happy in a strange way and was, oddly enough, dignified. It was poignant watching him leave in a wheelchair for what would be his last visit. He started sobbing and continuously waved at us as he rolled down the hallway disappearing into an elevator. I had never seen him as happy or weeping like that before. It was as if he were giving us his final salute and goodbye. As caregivers I am certain we all have had similar experiences.

While Captain Cordova’s poem made me recall that one patient, there have been others like him. It made me ponder the importance of compassionate understanding of patients’ dilemmas, and the doctor-patient relationship that is critical to success in providing health care in general, but particularly to those frustrating patients who are “Against Medical Advice.” I suppose the “angel eyes” of a caregiver, like a poet, can help us see the soul of patients, particularly those we can do little for because of noncompliance. We try, but sometimes we must simply accept patients’ autonomy with imperturbability and equanimity.
